# Non-Iatrogenic Localized-Reentrant Figure of Eight Atrial Tachycardias in the Superior Vena Cava

**DOI:** 10.1155/2023/5074946

**Published:** 2023-05-24

**Authors:** Shota Tokuno, Kenjiro Miyamoto, Ryuichi Usui

**Affiliations:** ^1^Cardiovascular Division, Department of Internal Medicine, Asahikawa Medical University, 2-1-1-1, Midorigaoka-Higashi, Asahikawa, Hokkaido 078-8510, Japan; ^2^Department of Cardiology, Sapporo Shiroishi Memorial Hospital, 8-Minami1-10, Shiroishi-ku, Sapporo, Hokkaido 003-0026, Japan

## Abstract

**Introduction:**

The superior vena cava (SVC) is an important non-pulmonary venous foci of atrial fibrillation (AF) and is known as the arrhythmogenic site of scar-related atrial tachycardia (AT). Scar-related ATs may occur after catheter ablation and open heart surgery; however, idiopathic AT rarely occurs. *Case Presentation*. A 77-year-old male with terminal diabetic nephropathy complained of dialysis-induced hypotension due to AF and was admitted to our hospital for catheter ablation. Here, we report a case of non-iatrogenic localized-reentrant figure of eight AT in the SVC.

**Conclusion:**

SVC has the arrhythmogenic potential for re-entrant tachycardia, and the development of mapping technology can reveal arrhythmogenic mechanisms.

## 1. Introduction

It is well known that scar-related atrial tachycardias (ATs) may occur after catheter ablation and open heart surgery [1]. The electrograms of ATs may show complex arrhythmias, and ablation can be challenging [2]. However, occasionally, a case of idiopathic AT arises. To the best of our knowledge, ATs originating from the superior vena cava (SVC) are rare, and the underlying mechanisms are unclear.

Currently, activation mapping technology is being developed. Three-dimensional mapping using a coherent map integrated with a vector and velocity map (CARTO®3 version 7; Biosense Webster Inc., Diamond Bar, CA, USA) helps to identify the mechanism of arrhythmia, allowing us to differentiate a focal source from a localized reentry [3, 4]. Here, we report a case of a non-iatrogenic localized-reentrant figure of eight AT in the SVC.

## 2. Case Presentation

**Figure 1:**
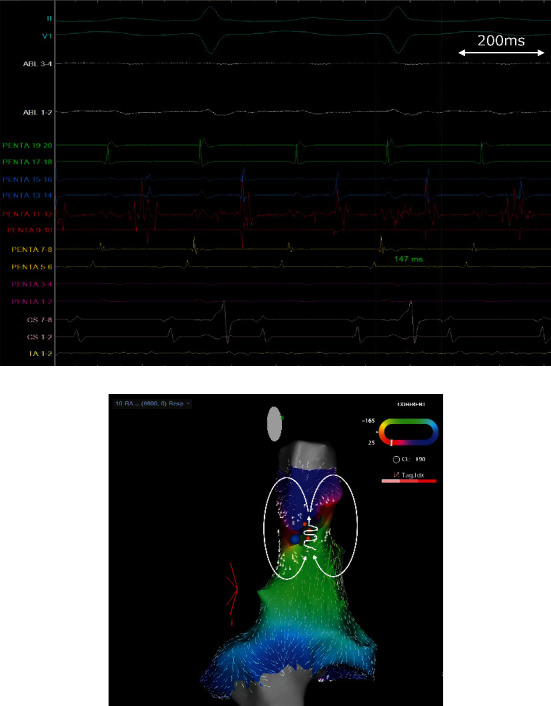
(a) Intracardiac electrogram recorded by the mapping catheter. This reveals the long duration of fragmented potentials at the slow conduction isthmus. (b) The activation map of the SVC–RA during AT shows curved vectors surrounding (white line). The focal blue point indicates the AT termination point, and the phrenic nerve sites were identified by pacing (orange points).

A 77-year-old man with terminal diabetic nephropathy complained of dialysis-induced hypotension due to atrial fibrillation (AF) and was admitted to our hospital for catheter ablation. He was receiving dialysis via the left hand's autologous arteriovenous fistula. We performed circumferential pulmonary vein isolation using a Thermo-Cool STSF catheter (Biosense Webster Inc.) under CARTO®3 version 7, and achieved bidirectional blocks of both pulmonary veins. After the procedure, 200 ms burst programmed stimulation of the atrium induced AT with a cycle length of 190 ms. The activation sequence of the AT demonstrated constantly from proximal to distal in the coronary sinus (CS) and from superior to inferior in the right atrium (RA), and early activations were located at the SVC site. The SVC and RA maps were acquired using a multielectrode mapping catheter (Pentaray; Biosense Webster Inc.) and the coherent map module. Electrical activation revealed localized reentry on the SVC, appearing as a double loop circuit or a figure of eight (Supplemental Videos 1 and Figures 1(a) and 1(b)). Moreover, slow conduction areas were located in the posterolateral SVC. There was a long duration and fractionated electrogram (147 ms, 77% of the tachycardia cycle length) at the entrance, and the post-pacing interval was nearly equal to the total cycle length at the site. Thus, AT was diagnosed as a localized reentrant AT of the de novo SVC. We identified the right phrenic nerve site by several pacings, and the radiofrequency energy terminated the AT near the entrance where the phrenic nerve was not captured. After the procedure, the SVC-RA connection was residual by CS pacing; therefore, we achieved circumferential SVC isolation, and SVC firing was detected. Thereafter, no atrial tachyarrhythmias were completely induced by programmed stimulation, and infusion of isoproterenol and adenosine triphosphate.

## 3. Discussion

The main findings of this case were the arrhythmogenic possibility of SVC and the efficacy of coherent mapping.

First, we described a patient with non-iatrogenic localized-reentrant AT on the SVC. SVCs are known to be the foci site of AF and the arrhythmogenic site of scar-related AT [5], and clearly found that the de novo SVC could be the circuit of the localized figure of eight AT in small areas.

**Figure 2:**
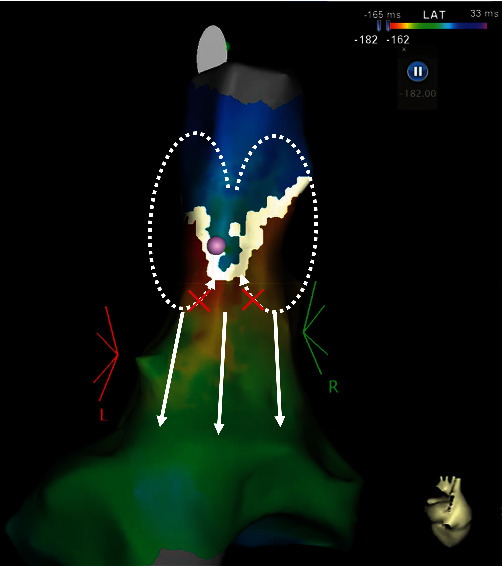
The conventional propagation map using local activation time annotation. This map demonstrates a focal AT propagating from the superior to inferior wall in the RA, and appears to be a block line at the slow conduction isthmus. The pink point indicates the termination point of the AT.

Second, we recognize AT as a localized reentry because a coherent map revealed the circuit of the AT and slow conduction isthmus. However, conventional activation mapping based on local activation time alone could not describe the slow conduction regions due to inaccurate annotation of fragmented electrograms, and the tachycardia appeared to be a focal AT (Supplemental Video 2, Figure 2).

The coherent map was useful for differentiating a localized reentrant mechanism from a true focal mechanism; thus, we may easily recognize the localized reentrant tachycardias that appear as focal tachycardias mapped by the conventional activation mapping system. A more developed mapping technology permits more frequent detection of localized-reentrant ATs in the future; thus, SVC-localized reentrant ATs may become less rare. Furthermore, continuous mapping technology development will continue to improve the recognition of arrhythmogenic mechanisms and contribute to enhanced procedural outcomes of AT ablation.

## Data Availability

The image and video data used to support the findings of this study are included within the article and supplementary information files.

## Supplementary Materials

Supplementary Materials Supplemental Videos 1 The activation map during atrial tachycardia (AT) shows a localized figure of eight AT using a coherent map in the CARTO 3 system.

Supplementary Materials Supplemental Videos 2 The activation map during atrial tachycardia (AT) is similar to a focal AT using a conventional propagation map in the CARTO 3 system.

## References

[1] Knecht S., Veenhuzen G., O’Neill M. D. (2009). Atrial tachycardias encountered in the context of catheter ablation for atrial fibrillation part II: mapping and ablation. *Pacing and Clinical Electrophysiology*.

[2] Schaeffer B., Hoffmann B. A., Meyer C. (2016). Characterization, mapping, and ablation of complex atrial tachycardia: initial experience with a novel method of ultra high-density 3D mapping. *Journal of Cardiovascular Electrophysiology*.

[3] Anter E., Duytschaever M., Shen C. (2018). Activation mapping with integration of vector and velocity information improves the ability to identify the mechanism and location of complex scar-related atrial tachycardias. *Circulation. Arrhythmia and Electrophysiology*.

[4] Vicera J. J. B., Lin Y.-J., Lee P.-T. (2020). Identification of critical isthmus using coherent mapping in patients with scar-related atrial tachycardia. *Journal of Cardiovascular Electrophysiology*.

[5] Miyazaki S., Hasegawa K., Ishikawa E. (2019). Scar-related atrial tachycardia within a short superior vena cava musculature sleeve. *Journal of Cardiovascular Electrophysiology*.

