# Supplementary material for: Non-Iatrogenic Localized-Reentrant Figure of Eight Atrial Tachycardias in the Superior Vena Cava
Source: Case Rep Cardiol. 2023 May 24;2023:5074946. doi: 10.1155/2023/5074946 (PMC10232176; doi:10.1155/2023/5074946)
Supplement: Supplementary Materials — Supplemental Videos 1 The activation map during atrial tachycardia (AT) shows a localized figure of eight AT using a coherent map in the CARTO 3 system. [file 5074946.f1.docx]

**Supplemental Videos 1**

The activation map during atrial tachycardia (AT) shows a localized figure of 8 AT using a coherent map in the CARTO 3 system.

<https://drive.google.com/file/d/1EqpSKtbZtbN6keUZ1bPHdHz4BveK315e/view?usp=sharing>
