# Supplementary material for: Non-Iatrogenic Localized-Reentrant Figure of Eight Atrial Tachycardias in the Superior Vena Cava
Source: Case Rep Cardiol. 2023 May 24;2023:5074946. doi: 10.1155/2023/5074946 (PMC10232176; doi:10.1155/2023/5074946)
Supplement: Supplementary Materials — Supplemental Videos 2 The activation map during atrial tachycardia (AT) is similar to a focal AT using a conventional propagation map in the CARTO 3 system. [file 5074946.f2.docx]

**Supplemental Videos 2**

The activation map during atrial tachycardia (AT) is similar to a focal AT using a conventional propagation map in the CARTO 3 system.

**https://drive.google.com/file/d/1fQeGpQl5bPq8gdidJUcgbm4OOrJYSK_W/view?usp=sharing**
